# The efficacy and safety of the short ragweed sublingual immunotherapy tablet MK-3641 is similar in asthmatic and nonasthmatic subjects treated for allergic rhinitis with/without conjunctivitis

**DOI:** 10.1186/1710-1492-10-S2-A17

**Published:** 2014-12-18

**Authors:** Jennifer Maloney, David I Bernstein, Jacques Hébert, Martha White, Robert Fisher, Thomas B Casale, Amarjot Kaur, Hendrik Nolte

**Affiliations:** 1 Merck & Co., Inc., Whitehouse Station, NJ, USA; 2 Bernstein Allergy Group, Cincinnati, OH, USA; 3 Centre de Recherche Appliquée en Allergie de Québec, Québec City, QC, Canada; 4 Institute for Asthma & Allergy, Wheaton, MD, USA; 5 Allergy Research & Care, Milwaukee, WI, USA; 6 Department of Medical Microbiology/Immunology, Creighton University Medical Center, Omaha, NE, USA

## Background

We conducted a post-hoc analysis of two short-ragweed sublingual immunotherapy tablet (SLIT-T) trials to investigate whether the subjects with allergic rhinitis with/without conjunctivitis (AR/C) and comorbid asthma reported different efficacy or safety events than those with AR/C and no asthma.

## Methods

Data from two trials evaluating the short-ragweed SLIT-T MK-3641 (*Ambrosia artemisiifolia*; Merck/ALK-Abelló) were pooled. Subjects with ragweed-pollen–induced AR/C were randomized to once-daily MK-3641 (6 or 12 Amb a 1-U doses) or placebo for approximately 52 weeks. Subjects with AR/C and stable asthma not requiring medium- or high-dose inhaled corticosteroids and ≥70% predicted FEV_1_ were eligible. Efficacy and safety outcomes were assessed in subjects with AR/C with/without reported asthma. Efficacy measurements included AR/C total combined score (TCS; combined symptom+medication scores); safety was assessed by reported adverse events (AEs).

## Results

Among subjects with AR/C and asthma receiving MK-3641 6 or 12 Amb a 1-U, TCS was reduced by 17% (−1.27; 95% CI: −3.48, 0.93; n=56) and 22% (−1.68; 95% CI: −3.69, 0.33; n=64), respectively, versus placebo (mean TCS=7.65; n=64) over the 15-day peak season. Among subjects without asthma receiving MK-3641 6 or 12 Amb a 1-U, TCS was reduced by 21% (−1.83; 95% CI: −2.84, −0.82; n=261) and 27% (−2.34; 95% CI: −3.33, −1.35; n=247), respectively, versus placebo (mean TCS=8.73; n=269). At least one treatment-related AE was experienced by 33%, 63%, and 65% of placebo and MK-3641 6 and 12 Amb a 1-U subjects with asthma, respectively, versus 24%, 54%, and 60% of subjects without asthma. No treatment-related serious or life-threatening AEs or hypersensitivity or systemic reactions were observed.

## Conclusions

The overall number of subjects with asthma was low and the data must be interpreted with caution. However, the SLIT-T treatment MK-3641 appeared to demonstrate similar efficacy and safety results in subjects with ragweed AR/C with or without asthma.

## Trial registration

ClinicalTrials.gov Identifiers: NCT00783198; NCT00770315

